# Simultaneous Determination of Six Compounds in Destructive Distillation Extracts of Hawthorn Seed by GC-MS and Evaluation of Their Antimicrobial Activity

**DOI:** 10.3390/molecules24234328

**Published:** 2019-11-27

**Authors:** Hongyu Rao, Peibo Li, Hao Wu, Chong Liu, Wei Peng, Weiwei Su

**Affiliations:** Guangdong Engineering & Technology Research Center for Quality and Efficacy Reevaluation of Post-Market Traditional Chinese Medicine, Guangdong Key Laboratory of Plant Resources, School of Life Sciences, Sun Yat-sen University, Guangzhou 510275, China; raohongyu1205@163.com (H.R.); lipb73@126.com (P.L.); wuhao8@mail.sysu.edu.cn (H.W.); lclchong@hotmail.com (C.L.); pweiyu929@126.com (W.P.)

**Keywords:** hawthorn seed, GC-MS, phenolic compounds, antimicrobial activity

## Abstract

Hawthorn seed can be used to produce various bioactive compounds through destructive distillation. In this study, an accurate and feasible analytical method based on a gas chromatography mass spectrometer (GC-MS) was developed for simultaneous determination of six major compounds (contributing to more than 3% in total peak area) in destructive distillation extracts of hawthorn seed collected at different temperatures ranging from 150 to 270 °C. Then, a broth microdilution method coupled with grey correlation analysis was engaged in the evaluation of their antimicrobial activities and the screening of primarily active compounds. Results indicate that the extract collected from 211 to 230 °C had the highest content of six major compounds (furfural, 2-methoxyphenol, 2-methoxy-4-methylphenol, 4-ethyl-2-methoxyphenol, 2,6-dimethoxyphenol, and 5-tertbutylpyrogallol) and the strongest antibacterial activity. Besides, 2,6-dimethoxyphenol was found to be a potential compound in inhibiting the growth of vaginitis pathogens. This study provided an optimum temperature for the destructive distillation of hawthorn seed, reducing the waste of energy, and saving the cost of production in the hawthorn industry.

## 1. Introduction

*Crataegus pinnatifida* Bunge, generally known as hawthorn, has been used as food and herbal medicine in China and Europe for centuries. Hawthorn has been shown to possess a lot of biological activities, including: Antioxidant, anti-inflammatory, and antimicrobial properties, etc. [1,2]. Hawthorn seed is an important by-product of the hawthorn processing industry [3]. Several studies have been carried out to investigate the chemical profile of hawthorn seed and lignin was found to be its primary constituent [4]. With fast pyrolysis, lignin could be degraded into a mass of phenolic monomers and oligomers, which possess multiple bioactivities [5]. Recently, hawthorn seed was developed into an antibacterial product with destructive distillation [6]. In a preliminary study, the chemical composition of the destructive distillation extract of hawthorn seed was profiled with gas chromatography mass spectrometer (GC-MS). A total of 92 compounds (including phenols, aldehydes, furans, etc.) were identified. Phenolic compounds were found to be essential components, accounting for 46.47% in total peak area. However, the exact contents of these compounds are still unknown.

Generally, the compositions of destructive distillation extracts are associated with the pyrolytic temperature [7,8]. As bioactivities are determined by processing conditions [9], there is no doubt that the antibacterial activities of the destructive distillation extract of hawthorn seed are related to pyrolytic temperature. Therefore, it is meaningful to evaluate the optimum temperature of pyrolysis to obtain the extract with enhanced antibacterial activities.

In this study, a novel method based on GC-MS was established to determine the content of major compounds (contributing to more than 3% in total peak area) in destructive distillation extracts of hawthorn seed, which were obtained in different temperature ranges. Furthermore, a microdilution method was engaged to evaluate the in vitro antimicrobial activities of these extracts, and grey correlation analysis was used to screen the potential antimicrobial compounds. This study was to explore the difference of extracts at different distillation temperatures, so as to find out the optimal retorting temperature and identify the primary active compounds with antibacterial activities.

## 2. Results and Discussion

In this study, the hawthorn seeds were pyrolized under N_2_ at temperatures in the range of 150 to 270 °C. The anhydrous methanol was chosen as the solvent because other substrates (such as hexane, methylene chloride, and water) could form a very compact emulsion preventing the analysis with GC-MS. Considering the boiling points of the target analytes, the heating rate of the developed GC method decreased with a relatively slow rate after 100 °C to better separate the phenolic compounds [10,11].

**Figure 1:**
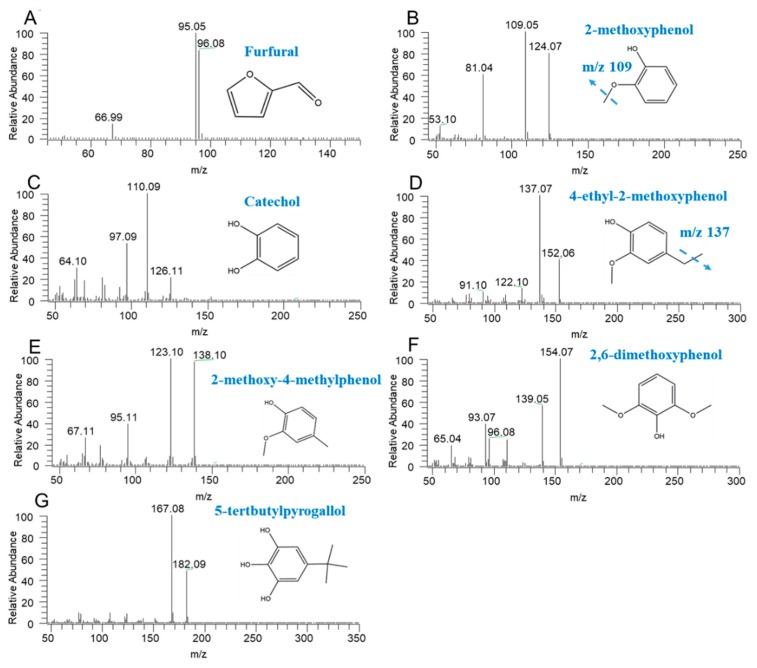
The characteristic ions of furfural (**A**), 2-methoxyphenol (**B**), catechol (**C**), 4-ethyl-2-methoxyphenol (**D**), 2-methoxy-4-methylphenol (**E**), 2,6-dimethoxyphenol (**F**), and 5-tertbutylpyrogallol (**G**).

As to the MS conditions, the peaks of the ion with the highest intensity was selected for the content determination. The MS details of those compounds are shown in Figure 1.

### 2.1. Method Validation

#### 2.1.1. Specificity

**Figure 2:**
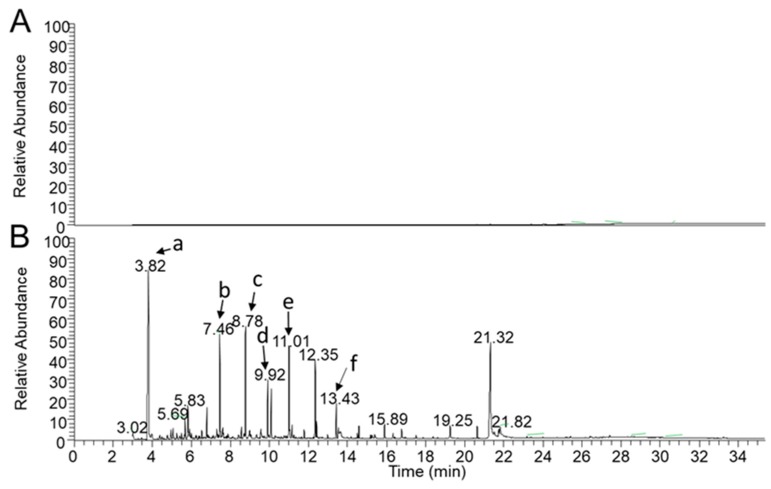
The total ion chromatogram (TIC) of methanol (**A**) and 150 °C–270 °C extract samples dissolved with methanol (**B**), peak a to f represented for furfural (**a**), 2-methoxyphenol (**b**), 2-methoxy-4-methylphenol (**c**), 4-ethyl-2-methoxyphenol (**d**), 2,6-dimethoxyphenol (**e**), and 5-tertbutylpyrogallol (**f**).

No instrumental carry-over or significant drift were noticed during all of the validation process. Compared with the reference standard, the mass spectrogram of the sample has no obvious interference peak. Meanwhile, no matrix effects were observed at the lowest level in the linear range. The total ion chromatogram (TIC) of the solvent and extract samples are shown in Figure 2. Therefore, this method can be used for content determination in this linear range.

#### 2.1.2. Linearity

The correlation coefficients (R) were always more than 0.99 indicating that there was a good correlation of linearity through the concentrations range level.

#### 2.1.3. Precision and Accuracy

The precisions and accuracies for furfural, 2-methoxyphenol, and catechol were within an acceptable range. The relative standard deviation (RSD) (Table 1) were <15% for all the analytes which indicates that the present method was reliable and reproducible for the simultaneous quantitative analysis of furfural, 2-methoxyphenol, and catechol in the extract samples with methanol as the solvent.

#### 2.1.4. Stability

All established stability tests exhibited acceptable values, the RSD (Table 1) for all analytes were <10% which indicated that all the analytes with methanol as the solvent were stabled in 48 h.

#### 2.1.5. Recovery

On the basis of recovery result of furfural, 2-methoxyphenol, and catechol ranged from 84.00–111.7%, 89.70–110.1%, and 93.66–106.7%, respectively (Table 1). The RSD for all analytes ranged from 4.25% to 9.44%, correspondingly.

### 2.2. Content Determination

A preliminary study about the composition of destructive distillation extracts showed that there are seven compounds contributing to more than 3% in total peak area, including: 2,6-dimethoxyphenol (9.79%), 1,2,4-trimethoxybenzene (9.32%), furfural (7.21%), 2-methoxy-4-methylphenol (5.36%), 5-tertbutylpyrogallol (5.33%), 2-methoxyphenol (4.41%), and 4-ethyl-2-methoxyphenol (4.41%). Among them, the phenolic compounds have the hydroxyl groups which could show potential abilities of free radical scavengers, suggesting that they could demonstrate antioxidant properties [12]. For example, the results of the 1,1-diphenyl-2-picrylhydrazyl (DPPH) test indicate that 2-methoxyphenol (guaiacol) could trap the DPPH radical [13]. Furthermore, the hydroxyl groups could also affect the proton exchange, thereby affecting the function of microbial membrane [14]. 4-ethyl-2-methoxyphenol could prevent the formation of biofilm, inhibiting the growth of bacterial [15]. Meanwhile, furfural with aldehydes in its structure also has multiple biological activities [16], such as giving rise to the formation of reactive oxygen species (ROS), causing damages to cell organelles, nucleic acids and proteins, and disturbing the evolution of microorganisms [17,18]. In addition, it could also inhibit enzymes in primary metabolism, such as ethanol dehydrogenase, which definitely affects the growth of yeast cells [19]. Thus, 1,2,4-trimethoxybenzene, without hydroxyl groups and aldehydes, was not considered when we started the content determination and the correlation analysis.

The result indicated that the concentration of major compounds in extracts from hawthorn seed range from 0.263 to 10.120 mg per g of dry extract, correspondingly. With the increase of temperature, the content of all compounds except furfural increased gradually in the range of 150 to 230 °C, then decreased after 230 °C, suggesting that the phenolic oligomers start to increase after the temperature exceeds 230 °C. Then, we added up the concentrations of the main compounds mentioned above and the result indicated that the total content of the major compounds in the extracts at 211 to 230 °C was higher than the other temperatures (Table 2).

### 2.3. Antimicrobial Activities

Extracts from plants, such as essential oils, are increasingly used as a preservative due to their safety and effectiveness against bacteria and fungi in food and drug industries [20]. Minimal inhibitory concentration (MIC) is a fast and effective way to detect whether the extracts have an inhibition effect on the growth of microorganisms [21]. Due to the different structures of cell walls, all related strains should be selected for a more comprehensive study of the antimicrobial activities [22,23]. In addition to that, the pathogen strains were always chosen to evaluate the efficacy of infectious diseases based on the clinical epidemiology [24,25]. Thus, five typical vaginitis pathogens (*Escherichia coli, Staphylococcus aureus, Pseudomonas aeruginosa, Gardnerella vaginalis*, and *Candida albicans*) covering gram-positive bacteria, gram-negative bacteria, and fungi were selected to determine the potential therapeutic effect on vaginitis of the extracts from hawthorn seed.

The MIC results for the in vitro antibacterial properties of the extracts from hawthorn seed range from 0.49 to 15.6 mg/mL, correspondingly (Table 3). All extracts showed moderate antibacterial activity against the tested strains suggesting that the extracts could be used to treat the infections caused by them, especially when it comes to vaginitis. Above all the anti-bacterial experiments, the MIC of the extracts at 211 to 230 °C was always smaller than the extracts collected from other temperatures, suggesting that its antibacterial activity was stronger than other extracts among these five strains, which may be affected by the highest content of main compounds. Meanwhile, the similar sensitivity to gram-positive bacteria and gram-negative bacteria suggests that the mechanism of the antimicrobial may be independent of the cell wall, but related to others, such as the function of the microbial membrane and direct injury to microbial organelles caused by ROS [26,27].

### 2.4. Grey Correlation Analysis

**Figure 3:**
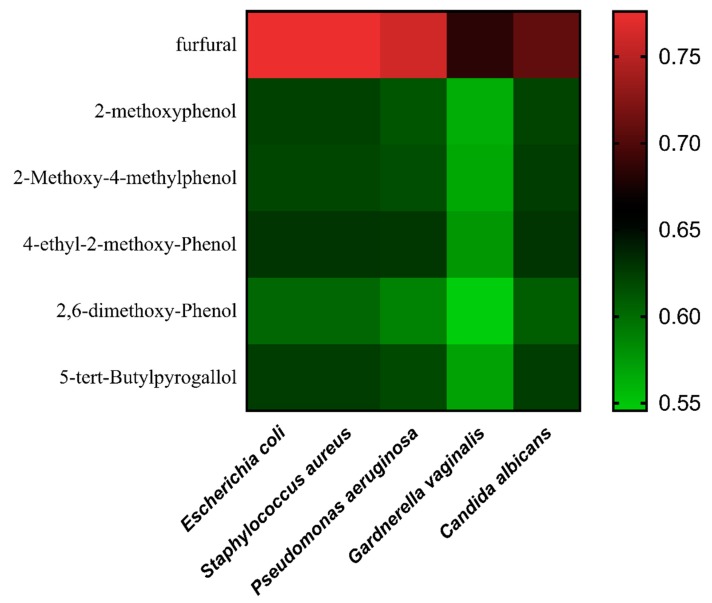
The heat map of the grey correlation analysis between the major compound concentration and the MIC.

In order to explore the effective antimicrobial substances in the extracts from hawthorn seed, the antimicrobial activity and the content of the main compounds among the different temperatures were studied by grey correlation analysis. The value of grey correlation analysis in this study range from 0.5456 to 0.7760, correspondingly. Different colors represent different ranges of values, and the greener the color is, the smaller the value (Figure 3). Then, the result indicated that 2,6-dimethoxyphenol plays an important role in inhibiting the growth of all test strains. This result is consistent with the study by Yang [28], suggesting that 2,6-dimethoxyphenol may be a potential monomer with antibacterial activity against the strains.

## 3. Materials and Methods.

### 3.1. Chemicals and Materials

Certified standards of furfural, 2-methoxyphenol, and catechol were purchased from Aladdin Industrial Corporation (Shanghai Aladdin Bio-Chem Technology Co. LTD, Shanghai, China). High-performance liquid chromatography (HPLC) grade methanol was purchased from Honeywell B&J (Ulsan, Korea).

The dried hawthorn seed was held for more than 30 min at 150 °C in a distillation kettle to remove the moisture and the volatile oils. Then, fractions were collected for every 20 °C until the temperature rose to 270 °C. The mix sample was collected from 150 to 270 °C, relatively (Table 4). All extracts used in this study were provided by Shandong Buchang Shenzhou Pharmaceutical Co. LTD (Heze, China).

The standard strains (*Escherichia coli* ATCC25922, *Staphylococcus aureus* ATCC25923, *Pseudomonas aeruginosa* ATCC27853) were provided by The First Affiliated Hospital of Sun Yat-sen University (Guangzhou, China). The other strains (*Gardnerella vaginalis* BNCC337545 and *Candida albicans* BNCC186382) were purchased from Bena culture collection (Suzhou Bena Chuanglian Biotechnology Co. LTD, Suzhou, China). The blood agar and the Sabauraud agar used for the passage of stains were purchased from BioMérieux (Lyon, France). Meanwhile, the Muller Hinton Broth and Sabouraud medium for test were procured from Hopebiol Corporation (Qingdao Hopebiol Biotechnology Co. LTD, Qingdao, China).

### 3.2. Preparation of Standards and Extract Samples

The stock solutions of furfural (20 mg/mL), 2-methoxyphenol (12 mg/mL), and catechol (14 mg/mL) were prepared in methanol. All stock solutions were stored at 4 °C and brought to room temperature before use. Working standards were diluted from stock solutions with methanol by 10 times to 2 times.

An aliquot of 3 mL extract was diluted into 10 mL with methanol, filtrated with a 0.22 μm microporous membrane, and then transferred into a 200 μL GC vial for GC analysis.

### 3.3. Gas Chromatography Mass Spectrometric Assay

The destructive distillation extracts of hawthorn seed were analyzed by GC-MS to identify the number and the contents of the compounds. A Thermo Fisher Trace 1300 gas chromatograph (Thermo Fisher Scientific, Milan, Italy) and a TSQ8000 mass spectrometer (Thermo Fisher Scientific, Milan, Italy) in trace ion detection mode with a programmable temperature injector were used to characterize secondary metabolites and aromatic compounds. The chromatographic separation was done on a capillary column of fused silica TG-5SilMS (30 m × 0.25 mm × 0.25 μm film thickness) provided by a Thermo Fisher Scientific (Milan, Italy) with helium as the carrier gas at a constant flow of 2.0 mL/min. The extracts (10 μL) were injected in the split mode (1:50) by empty baffled liner at 240 °C. The initial temperature was 40 °C and held for 3 min, rising to 100 °C at a heating rate of 15 °C/min, then rising to 270 °C at 8 °C/min and holding for 7 min. The temperatures of the injector and detector were 280 °C and 300 °C, respectively. Elements were scanned at 70 eV in electron ionization (EI) mode. And the mass spectra were acquired using the full-scan-monitoring mode, with the scan range of *m*/*z* 45–650. Then, the following characteristic ions were selected for quantification: *m*/*z* 95 for furfural, *m*/*z* 109 for 2-methoxyphenol, *m*/*z* 110 for catechol, *m*/*z* 137 for 4-ethyl-2-methoxyphenol, *m/z* 138 for 2-methoxy-4-methylphenol, *m*/*z* 154 for 2,6-dimethoxyphenol, and *m*/*z* 167 for 5-tertbutylpyrogallol, respectively. And, the dates were analyzed by the TraceFinder (v.4.1, Thermo Fisher Scientific).

### 3.4. Method Validation

Validation was achieved according to the Chinese Pharmacopoeia Commission in 2015.

Specificity was evaluated by checking that chromatographic peaks did not confuse any compounds with methanol. The evaluation consisted of verifying that the specific retention times and SIM responses of extracts were well discriminated from the substrate solution.

A calibration curve was constructed with five concentration levels of standard samples covering the range of 10–10,000 μg/mL. Each concentration level was prepared in duplicate. Meanwhile, the peak area was derived from the characteristic ions of each analytes. Furthermore, the content of each was determined by a linear equation with characteristic ions. Meanwhile, those without standards were treated with equations of structural analogues. For example, 5-tertbutylpyrogallol was determined using the linear method established by catechol because they all had multiple phenolic hydroxyl groups. The others determination was based on the linear method established by 2-methoxyphenol due to the methoxyl in the structures. Calibration curves were constructed and fitted by a linear least-squares regression analysis to plot the peak area against the concentrations.

Precision and accuracy were evaluated by repeated standards and mixed samples (*n* = 6) at concentrations of 28–6500 μg/mL on the same days.

To check the stability of the compounds, degradation tests were carried out 5 times in 48 h.

Recovery of analyses were assessed at medium concentration levels with 6 replicates.

Then, after all the samples’ analysis using freshly prepared calibration curves, imprecision, and accuracy were expressed by the RSD.

### 3.5. Determination of the Antimicrobial Activities

A broth microdilution method was engaged for the determination of the antimicrobial activities of the extracts from hawthorn seed which complied with the Clinical Laboratory and Standards Institute (CLSI, 2018) [29]. The test of *Candida albicans* was performed in Sabouraud medium (SDA), while the others were all performed in Muller Hinton Broth (MHB). A serial doubling dilution of the extracts was prepared in 96-well plates ranging from 0.24–62.5 mg/mL and the final concentration of microbial suspensions was 5 × 10^5^ CFU/mL (passage more than 2 times and stable growth). The MIC of the gardnerella was read after a 48-h-incubation at 37 °C while the other MIC was taken after 24 h. All determinations were performed in triplicate.

### 3.6. Grey Correlation Analysis

The grey correlation analysis was based on the similar degree of geometric shape of the data series curve, which can be used to process small amounts of irregular data [30]. If the curve is closer, the correlation among the relative data will be better. In this study, the contents of phenolic compounds and furfural in different extracts were taken as the independent variable, while the MIC of different extracts were taken as the dependent variable. If the value of grey correlation analysis is lower, the MIC of the compound against the strain will be smaller, which indicates that it has stronger antibacterial activity.

## 4. Conclusions

In the present study, an accurate and feasible analytical method based on GC-MS was established for simultaneous determination of six major compounds in destructive distillation extracts of hawthorn seed collected at different temperatures ranging from 150 to 270 °C. Furthermore, the optimum extraction temperature was investigated based on the content of the main components and antibacterial activities. Obtained results revealed that the extracts collected at 211 to 230 °C had the strongest antibacterial activity and the highest content of main compounds, suggesting that 211 to 230 °C is the optimum extraction temperature for the destructive distillation of hawthorn seed. Moreover, the result from the grey correlation analysis indicates that 2,6-dimethoxyphenol is a monomer with potential antibacterial activity, deserving better development and utilization. This work would be useful for the distillation of hawthorn seed in industrial manufacture.

## Figures and Tables

**Table 1:** The precision, stability, and recovery of furfural, 2-methoxyphenol, and catechol in the extracts from hawthorn seed.

Compound	Precision (*n* = 6)		Stability (*n* = 5)		Recovery (%) (*n* = 6)	Recovery RSD (%)
	Standards	Extracts	Standards	Extracts		
furfural	6.15%	4.38%	6.61%	7.47%	84.00–111.7%	9.44%
2-methoxyphenol	2.06%	4.08%	3.69%	5.93%	89.70–110.1%	6.91%
catechol	10.53%	6.76%	6.28%	5.45%	93.66–106.7%	5.10%

**Table 2:** The concentration of major compounds in the extracts from hawthorn seeds.

Compound	Concentration (mg/g)						
	150–170 °C	171–190 °C	191–210 °C	211–230 °C	231–250 °C	251–270 °C	150–270 °C
furfural	4.196	10.120	9.009	7.743	6.410	8.400	5.835
2-methoxyphenol	0.480	0.948	2.995	5.497	6.034	3.960	1.303
2-methoxy-4-methylphenol	0.387	0.889	2.792	5.569	5.660	3.322	1.092
4-ethyl-2-methoxyphenol	0.263	0.655	2.018	4.091	3.637	2.103	0.853
2,6-dimethoxyphenol	0.582	1.182	4.890	8.969	9.360	5.983	1.441
5-tertbutylpyrogallol	0.773	1.573	5.103	10.010	8.913	5.738	2.134

**Table 3:** The in vitro antibacterial of the extracts from hawthorn seed against *Escherichia coli*, *Staphylococcus aureus*, *Pseudomonas aeruginosa*, *Gardnerella vaginalis*, and *Candida albicans*.

Extract Temperature (°C)	Minimal Inhibitory Concentration (MIC) in mg/mL				
	*Escherichia Coli*	*Staphylococcus Aureus*	*Pseudomonas Aeruginosa*	*Gardnerella Vaginalis*	*Candida Albicans*
150–170	3.90	3.90	1.95	7.81	15.6
171–190	1.95	1.95	0.98	3.90	7.81
191–210	0.98	0.98	0.98	3.90	1.95
211–230	0.98	0.98	0.49	1.95	0.98
231–250	0.98	0.98	0.98	3.90	1.95
251–270	0.98	0.98	0.98	3.90	3.90
150–270	0.98	0.98	0.98	7.81	1.95

**Table 4:** The experimental details about the process of the dried hawthorn seed.

The Dose of Dried Hawthorn Seed (kg)	Extract Temperature (°C)	The Volume of the Extracts (L)
5616	150–170	110
	171–190	170
	191–210	400
	211–230	400
	231–250	120
	251–270	170
	150–270	1370
